# Participation of 14-3-3ε and 14-3-3ζ proteins in the phagocytosis, component of cellular immune response, in *Aedes* mosquito cell lines

**DOI:** 10.1186/s13071-017-2267-5

**Published:** 2017-08-01

**Authors:** Abel Trujillo-Ocampo, Febe Elena Cázares-Raga, Rosa María del Angel, Fernando Medina-Ramírez, Leopoldo Santos-Argumedo, Mario H. Rodríguez, Fidel de la Cruz Hernández-Hernández

**Affiliations:** 1 Departamento de Infectómica y Patogénesis Molecular, Centro de Investigación y de Estudios Avanzados del Instituto Politécnico Nacional (CINVESTAV-IPN), Ciudad de México, Mexico; 2 Departamento de Biomedicina Molecular, Centro de Investigación y de Estudios Avanzados del Instituto Politécnico Nacional (CINVESTAV-IPN), Ciudad de México, Mexico; 3 Centro de Investigación Sobre Enfermedades Infecciosas, Instituto Nacional de Salud Pública, Cuernavaca, Morelos Mexico

**Keywords:** Aag-2, *Ae. aegypti*, C6/36 HT, *Ae. albopictus*, 14-3-3ε, 14-3-3ζ, DsiRNA, 14-3-3 inhibitor, Phagocytosis

## Abstract

**Background:**

Better knowledge of the innate immune system of insects will improve our understanding of mosquitoes as potential vectors of diverse pathogens. The ubiquitously expressed 14-3-3 protein family is evolutionarily conserved from yeast to mammals, and at least two isoforms of 14-3-3, the ε and ζ, have been identified in insects. These proteins have been shown to participate in both humoral and cellular immune responses in *Drosophila*. As mosquitoes of the genus *Aedes* are the primary vectors for arboviruses, causing several diseases such as dengue fever, yellow fever, Zika and chikungunya fevers, cell lines derived from these mosquitoes, Aag-2 from *Aedes aegypti* and C6/36 HT from *Aedes albopictus*, are currently used to study the insect immune system. Here, we investigated the role of 14-3-3 proteins (ε and ζ isoform) in phagocytosis, the main cellular immune responses executed by the insects, using *Aedes* spp. cell lines.

**Results:**

We evaluated the mRNA and protein expression of 14-3-3ε and 14-3-3ζ in C6/36 HT and Aag-2 cells, and demonstrated that both proteins were localised in the cytoplasm. Further, in C6/36 HT cells treated with a 14-3-3 specific inhibitor we observed a notable modification of cell morphology with filopodia-like structure caused through cytoskeleton reorganisation (co-localization of 14-3-3 proteins with F-actin), more importantly the decrease in *Salmonella typhimurium*, *Staphylococcus aureus* and *E. coli* phagocytosis and reduction in phagolysosome formation. Additionally, silencing of 14-3-3ε and 14-3-3ζ expression by mean of specific DsiRNA confirmed the decreased phagocytosis and phagolysosome formation of pHrodo labelled *E. coli* and *S. aureus* bacteria by Aag-2 cells.

**Conclusion:**

The 14-3-3ε and 14-3-3ζ proteins modulate cytoskeletal remodelling, and are essential for phagocytosis of Gram-positive and Gram-negative bacteria in *Aedes* spp. cell lines.

**Electronic supplementary material:**

The online version of this article (doi:10.1186/s13071-017-2267-5) contains supplementary material, which is available to authorized users.

## Background

Phagocytosis by haemocytes is the main innate immune process to eliminate invading bacteria in insects [1–4]. This process is initiated with the recognition of the foreign particle, followed by remodelling of the phagocytic cell cytoskeleton and plasma membrane invagination resulting in internalisation of particles into a phagosome [5]. Phagosomes then undergo serial fusions with endosomes and lysosomes (maturation process), to form a fully functional phagolysosome, where degradation and destruction of ingested particles occur [6]. Several intracellular signalling pathways that drive the insect immune system are similar to those of the mammalian innate immune system [7, 8]. Phagocytosis, a major innate immune process in insects, is crucial for tissue homoeostasis and remodelling since it is required for clearance of apoptotic bodies during embryogenesis and metamorphosis where tissues are remodelled, and cells under programmed death have to be removed [8].

The interference RNA (RNAi) based screening strategy has been used to identify proteins participating in phagocytosis of Gram-negative and Gram-positive bacteria by *Drosophila* hemocyte S2 cells. The evolutionally conserved protein 14-3-3ζ was found to contribute to bacterial engulfment and microbial resistance in insects [3, 9]. The 14-3-3 proteins in eukaryotic cells are a group of conserved acidic proteins that bind phosphoserine/phosphothreonine motifs. Seven 14-3-3 isotypes (α/β, γ, τ/θ, ε, η, σ, ζ/δ), encoded by individual genes, have been identified in mammals [10] and two (ε and ζ) in insects, such as *Drosophila*, *Bombyx mori* and *Aedes aegypti* [11–13]. 14-3-3 proteins are also scaffolding proteins that interact with many protein partners to regulate signalling pathways and control cytoskeleton remodelling through the binding of actin molecules, the essential element in phagocytosis [3, 14–16].

*Aedes* spp. mosquitoes are vectors of disease-causing arboviruses such as dengue, yellow fever, chikungunya and Zika [17, 18]. In a previous study, we identified two, 14-3-3ε and 14-3-3ζ isoforms in *Ae. aegypti*, that were highly homologous to the corresponding *Drosophila* orthologues, suggesting that they may have conserved functional roles in phagocytosis [13]. In this work, we investigated the role of the 14-3-3 isoforms in phagocytosis of Gram-positive and Gram-negative bacteria in the two cell-lines Aag-2 derived from *Ae. aegypti* and C6/36 HT from *Ae. albopictus*.

## Methods

### Cell culture

C6/36 HT cells from *Ae. albopictus* [19], adapted to grow at 34 °C, were cultured in minimum essential medium (Gibco, Thermo Fisher Scientific, Waltham, Mass, USA) supplemented with 7% fetal calf serum, 0.370 g/l sodium bicarbonate and 50 U/ml of penicillin and 50 μg/ml of streptomycin [19, 20]. *Ae. aegypti* Aag-2 cells (kindly provided by Dra. Isabel Salazar from Instituto Politécnico Nacional) were maintained at 28 °C in Schneider’s *Drosophila* medium with L-glutamine (Gibco, Thermo Fisher Scientific) supplemented with 10% FCS (Gibco, Thermo Fisher Scientific) adding 50 U/ml of penicillin and 50 μg/ml of streptomycin; the cells were released from the culture flask with trypsin-EDTA (0.05%) [20].

### Cell viability study

To evaluate cell viability in the presence of 14-3-3 inhibitor (Antagonist I, 2–5) [21, 22], 8 × 10^4^ cells were grown in 96 well plates (2500 cells/mm^2^) until they reached the exponential phase [23]. The first 15 h the cells remain in the Lag phase of growth. Subsequently, they enter the Log phase of growth; we performed the cell viability at 24 h. Subsequently, cells were gently washed with serum-free medium. The cells were incubated 120 min at 34 °C with several concentrations of 14-3-3 inhibitor (dissolved in DMSO) (12.5, 25, 50 and 100 μM), DMSO (vehicle used to dissolve the inhibitor) (Sigma-Aldrich, St. Louis, MO, USA) and medium (without inhibitor) in 100 μl of fresh serum free medium. Cells were then incubated with CellTiter96® AQueous One Solution Reagent for 60, 120 and 180 min at 34 °C [23, 24] (Promega Corporation, Madison, WI, USA) according to the manufacturer’s protocol. The experiments were performed in triplicate.

### Reverse transcriptase polymerase chain (RT-PCR) analysis

Briefly, total RNA was isolated from C6/36 HT and Aag-2 cells using Trizol (Invitrogen, Life Technologies, CA, USA), according to the manufacturer’s instructions, and treated with TurboDNase (Thermo Scientific, Waltham, Mass, USA). To synthesise the first strand of cDNA 500 ng of total RNA was used using oligo (dT) primers and SuperScript II reverse transcriptase (Invitrogen, Life Technologies, CA, USA), according to the manufacturer’s protocol. Finally, 14-3-3ε and 14-3-3ζ transcripts from C6/36 HT and Aag-2 cells were amplified by-PCR using specific primers sets for *Aeae*14-3-3Ɛ (*Ae. aegypti*, AAEL011116) and *Aeae*14-3-3ζ (*Ae. aegypti*, AAEL006885) [13]. For PCR amplification samples were preheated at 95 °C for 4 min, cycling conditions consisted on denaturing at 94 °C for 1 min, annealing at 58 °C (*Aeal*14-3-3Ɛ) or 60 °C (*Aeal*14-3-3ζ and S7) for 1 min for thirty-five cycles and a final extension step at 72 °C for 1 min. The *Aeal*14-3-3Ɛ and *Aeal*14-3-3ζ PCR products were sequenced (3500xL Genetic Analyser, Applied Biosystems, Life Technologies) and showed 100% match with the known sequences of *Aeae*14-3-3Ɛ and *Aeae*14-3-3ζ, respectively (data not shown).

### Western blot

Aag-2 and C6/36 HT cells were washed twice with phosphate buffered saline (PBS) and lysed in Radio-Immunoprecipitation Assay (RIPA) buffer (25 mM Tris, 150 mM NaCl, 0.1% SDS, 1% Triton X100) containing Complete™ protease inhibitors cocktail (Roche Diagnostics, Indianapolis, Ind, USA) and PhosSTOP™ phosphatase inhibitor cocktail (Roche Diagnostics). Cell lysates were then centrifuged at 16,000× *g* at 4 °C for 12 min, and supernatants were used immediately or stored at -80 °C until use. Protein was quantified in the samples using the BCA protein assay (Thermo Scientific). For Western blot, 30 μg of protein per lane were resolved in duplicate in 15% SDS-PAGE, one gel was stained with Coomassie blue and the other electrotransferred onto nitrocellulose (NC) membranes. After blocking with 5% bovine serum albumin (BSA) in TBS-T, membranes were incubated with rabbit polyclonal to human 14-3-3 (anti-14-3-3 K19 SC-629, dilution 1:1000; Santa Cruz Biotechnology, Inc., Dallas, TX, USA) overnight at 4 °C. This antibody is suitable to recognise 14-3-3ε and 14-3-3ζ proteins in *Ae. aegypti* mosquito in dilution of 1:1000 [13], followed by a secondary antibody goat anti-IgG Rabbit-HRP conjugated, (dilution 1: 80,000, Chemicon International, Billerica, Mass, USA). Signals detection was performed using the Supersignal West Pico Chemiluminescent Kit (Thermo Scientific) and an ImageQuant LAS 4000 System (GE Healthcare Life Sciences, Pittsburgh, PA, USA). For Western blotting, actin was used as loading control, NC membranes were stripped using Western Blot Stripping Buffer (Thermo Scientific), and incubated with an anti-actin mouse monoclonal antibody in dilution of 1: 40,000 (Abcam, Cambridge, UK), followed by a goat anti-Mouse IgG-HRP (dilution 1: 50,000, AP308P, Chemicon International). Signal detection was performed as described above.

### Immunofluorescence assays

C6/36 HT cells that were grown to 60% confluence on coverslips were incubated either with serum-free medium (SFM) alone, SFM with DMSO, or SFM with 100 μM of 14-3-3 inhibitor for 120 min at 34 °C. The cells were fixed with 4% paraformaldehyde for 30 min at room temperature, following by permeabilization with PBS containing 0.1% Triton X-100 for 10 min. After blocking with PBS added of 10% FCS, 3% BSA, 10 mM glycine for 60 min at 37 °C, cells were incubated with rabbit polyclonal anti-14-3-3 (clone K19, 1:100 dilution) and anti-Rabbit IgG Alexa Fluor 647 conjugate (Cell Signaling Technology, Danvers, Mass, USA) as secondary antibody, then cells were incubated with Rhodamine-Phalloidin (ThermoFisher Scientific) for 20 min. DAPI (H-1200, VECTASHIELD, Vector Laboratories Inc., Burlingame, CA, USA) was used to stain nuclei. Fluorescence was visualised under a Zeiss LSM 700 confocal microscope, and images were processed with the ZEN 2009 Light Edition software (Carl Zeiss, Oberkochen, Germany).

### Phagocytosis study

To evaluate phagocytosis, FITC labelled *Escherichia coli* [25], and *Salmonella typhimurium*-GFP [26] were incubated with C6/36 HT cells in a 10:1 ratio for 45 min at 34 °C or 4 °C (negative control). Mosquito cells were detached by scraping and resuspended in ice-cold PBS. Engulfed bacteria recorded by flow cytometry in a Cyan ADP Analyzer (Beckman Coulter, Inc., Indianapolis, IN, USA). The fluorescence intensity was acquired in the FL1 channel (FITC/GFP). To evaluate phagosome formation, we utilised pHrodo green Bio Particles - *E. coli* or *Staphylococcus aureus* labelled with the acid sensor pHrodo-succinimidyl ester (pHrodo™ SE Green, Life Technologies). This dye is activated in acid cell compartments such as phagolysosome [27, 28]. Lyophilized pHrodo green BioParticles were reconstituted in Uptake Buffer (20 mM HEPES in HBSS Hank’s balanced salt solution, pH 7.4, ThermoFisher) at a concentration of 1 mg/ml prior to use, 50 μl of resuspended pHrodo green BioParticles were added to either C6/36 HT cells cultured with 14-3-3 inhibitor or 14-3-3 silenced Aag-2 cells in 24 wells plates for 120 min. Then, cells were detached, single-cell resuspended and subjected to flow cytometric analysis of fluorescence intensity of pHrodo green. The fluorescence intensity was acquired in the FL1/FL2 channels (pHrodo green-509/540 nm). The cytometry result was expressed as mean fluorescence intensity (MFI), and phagocytosis percentage and phagocytosis percentage of inhibition [25].

### Knockdown of 14-3-3Ɛ and 14-3-3ζ proteins by DsiRNAs

27mer Dicer-substrate short interfering RNAs (DsiRNAs) were used to silence 14-3-3 expression. Target sites on DsiRNA14-3-3Ɛ (AAEL006885) and DsiRNA14-3-3ζ (AAEL011116) were designed using software provided in the Integrated DNA Technologies site (IDT; https://www.idtdna.com/site) following the provider’s instructions (Table 1). Briefly, 100 pmol of DsiRNA14-3-3Ɛ, DsiRNA14-3-3ζ or a negative DsiRNA were incubated with 1.5 μl of Lipofectamine™ RNAiMAX in 24-well tissue culture plates for 20 min at room temperature. Then, 3.9 × 10^5^ Aag-2 or C6/36 HT cells were added per well, mix gently and incubated at 28 °C and 34 °C, respectively. At 24 h posttransfection, the medium was removed and a second transfection procedure was performed to enhance knockdown efficiencies. After double transfection, the cells were cultured for additional 72 h, and the silencing of 14-3-3 proteins was confirmed from cell lysates using Western blotting as described above.

**Table 1:** Templates for DsiRNA generation designed for 14-3-3 silencing. DsiRNAs were synthesised using the custom Dicer-substrate siRNA Construction Kit (IDT, USA). RNA duplex has an asymmetric design with a single 2-base 3′-overhang on the antisense strand and is blunt on the other end; the blunt end is modified with DNA bases marked in bold

	Sequence (5′-3′)
DsiRNA14-3-3ε	
DsiRNA duplex1For	CAGUCUUCUACUACGAGAUCCUA**AA**
DsiRNA duplex1Rev	UUUAGGAUCUCGUAGUAGAAGACUGAG
DsiRNA duplex2For	GGGAGAUUACUACAGAUACCUAG**CC**
DsiRNA duplex2Rev	GGCUAGGUAUCUGUAGUAAUCUCCCUU
DsiRNA duplex3For	GGGAAAUCUGCUACGAAGUAUUG**GG**
DsiRNA duplex3Rev	CCCAAUACUUCGUAGCAGAUUUCCCUA
DsRNA14-3-3ζ	
DsiRNA duplex1For	GGUCAACAAGACAUAGCAAUUGG**AA**
DsiRNA duplex1Rev	UUCCAAUUGCUAUGUCUUGUUGACCUC
DsiRNA duplex2For	CAGUGACAUCGCAAUGACUGACC**TT**
DsiRNA duplex2Rev	AAGGUCAGUCAUUGCGAUGUCACUGGC
DsiRNA duplex3For	UCAGCUGAGUGGUGUAUAAAGAG**AA**
DsiRNA duplex3Rev	UUCUCUUUAUACACCACUCAGCUGAUU
DsRNANeg	
DsiRNANegFor	Phos-CUUCCUCUCUUUCUCUCCCUUGUGA
DsiRNANegRev	UCACAAGGGAGAGAAAGAGGAAGGA

### Statistical analysis

GraphPad Prism statistical software package (Prism 5.0; Graph-Pad Software, Inc., San Diego, CA) was used to perform statistical analysis. All data were presented as the mean ± SEM (standard error of the mean). The statistical comparisons of data between the different conditions were performed using one-way analysis of variance (ANOVA) or Student’s *t*-test. *P*-values below 0.05 were considered statistical significance.

## Results

### *Aedes* spp. cells express 14-3-3ε and 14-3-3ζ proteins

**Fig. 1:**
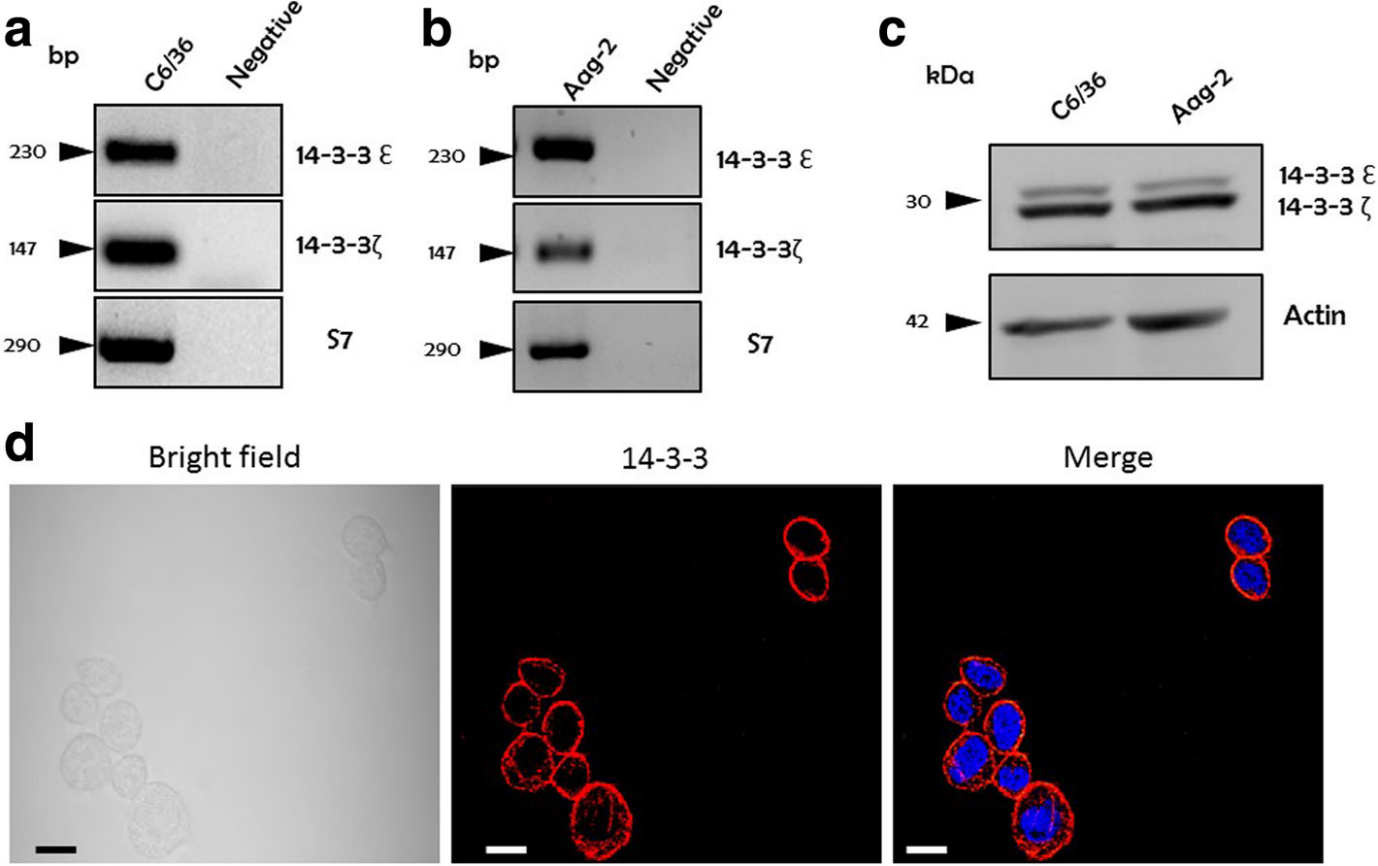
Expression of the 14-3-3 isoforms in C6/36 HT and Aag-2 cells. **a**, **b** The mRNA expression of 14-3-3ε and 14-3-3ζ. RT-PCR was performed to evaluate the presence of 14-3-3 mRNA transcript from C6/36 HT and Aag-2 cells, confirmed that both cell lines expressed 14-3-3ε and 14-3-3ζ isoforms at the mRNA level. S7 mRNA was used as an internal control. **c** 14-3-3 protein expression in C6/36 HT and Aag-2 cells. Cell lysates were assessed for the presence of 14-3-3 proteins by Western blot, which demonstrated two distinctive bands with the predicted sizes of 30 and 29 kDa corresponding to 14-3-3Ɛ and 14-3-3ζ protein isoforms, respectively in C6/36 HT and Aag-2 cells. **d** Intracellular distribution of 14-3-3 proteins in C6/36 HT cells. The immunofluorescent staining of 14-3-3 proteins reveals the presence of the 14-3-3 proteins (*red*) in the cytoplasm of C6/36 HT cells. Nuclei were stained with DAPI (*blue*). *Scale-bars*: 10 μm

We first evaluated the gene expression of *Aeae*14-3-3ε and *Aeae*14-3-3ζ in cell lines derived from *Ae. aegypti* and *Ae. albopictus*. RT-PCR amplifying specific segments of 14-3-3ε and 14-3-3ζ transcripts confirmed that they were highly expressed in C6/36 HT and Aag-2 cells (Fig. 1a, b). In addition, Western blot assays using anti-14-3-3 antibody (clone K-19) demonstrated two bands with the predicted molecular weights of ~30 and ~29 kDa corresponding to 14-3-3ε and 14-3-3ζ protein isoforms, respectively, in both Aag-2 and C6/36 HT cells (Fig. 1c). The abundance in the expression of the two 14-3-3 isoforms was similar in the two cell lines (Fig. 1c). Lastly, intracellular distribution of 14-3-3 proteins was assessed using Immunofluorescence assay as 14-3-3 protein family interacts with other proteins and may have a distinct pattern of intracellular expression [29, 30]. The 14-3-3 proteins, in basal conditions, were in the cytoplasm, mainly with a cortical distribution (Fig. 1d).

### Effect of 14-3-3 inhibitor on C6/36 HT cells phagocytosis

**Fig. 2:**
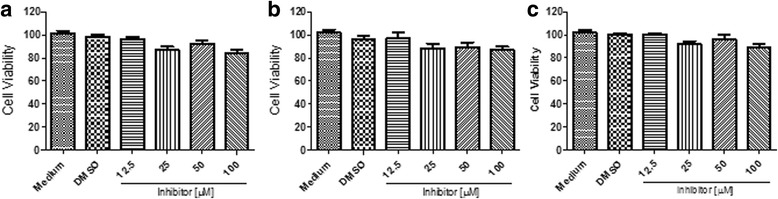
Effect of the 14-3-3 inhibitor on the viability of C6/36 HT cells. C6/36 HT cells were treated with various 14-3-3 inhibitor concentrations ranging from 12.5 μM to 100 μM for different times: at 60 min (**a**), 120 min (**b**) and 180 min (**c**), and cell viability was assessed. DMSO and medium alone were used as controls. Even prolonged treatment with 14-3-3 inhibitors, cell viability, was maintained at greater than 90%. Experiments were performed in triplicate; three independent experiments were conducted and represented as the mean ± standard error, SE

**Fig. 3:**
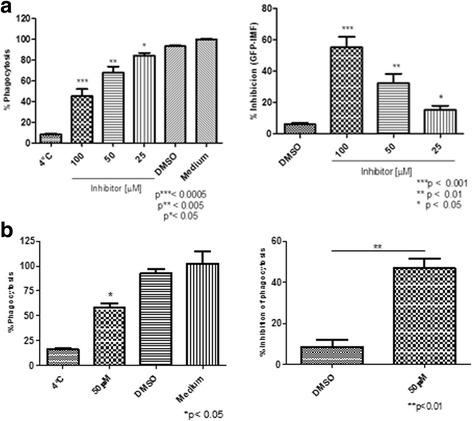
Effect of the 14-3-3 inhibitor on the phagocytosis of *S. typhimurium*-GFP and FITC-labeled *E. coli* in C6/36 HT cells. C6/36 HT cells pre-treated with 14-3-3 inhibitor at various concentrations were evaluated for phagocytosis of *S. typhimurium*-GFP (**a**) and FITC-labeled *E. coli* (**b**). Phagocytosis of both *S. typhimurium* and *E. coli* was significantly decreased by the treatment with 14-3-3 inhibitors in a dose-dependent manner. DMSO and medium alone, as well as phagocytosis performed at 4 °C, were used as negative control. Experiments were performed in triplicate, and data from three experiments are presented as the mean ± standard error, SE

To investigate the role of 14-3-3 in C6/36 HT cells functions, we assessed whether 14-3-3 inhibitor affects the cell viability, and found that viability was maintained at 90% even with highest concentration used (100 μM) and prolonged incubation time (180 min) (Fig. 2). In addition, we also evaluated whether the inhibitor induced disruption of protein-protein interaction with 14-3-3 had impact on phagocytosis. The treatment with 14-3-3 inhibitor in different concentrations ranging from 12.5 μM to 100 μM showed a significant reduction on phagocytosis of *S. typhimurium*-GFP, of 60% (Kruskal-Wallis test: *χ* ^2^ = 10.38, *df* = 5, *P* = 0.015; t-test: *t* = 7.318, *P* = 0.0019), 40% (Kruskal-Wallis test: *χ* ^2^ = 10.38, *df* = 5, *P* = 0.015; t-test: *t* = 4.266, *P* = 0.01) and 20% (Kruskal-Wallis test: *χ* ^2^ = 10.38, *df* = 5, *P* = 0.015; t-test: *t* = 3.277, *P* = 0.030) of inhibition at concentrations of 100 μM, 50 μM and 25 μM of 14-3-3 inhibitor, respectively (Fig. 3a). Comparable results were obtained in phagocytosis of FITC-*E. coli*, leading to a statistically significant decrease by up to 60% (t-test: *t* = 2.941, *P* = 0.0022), with 50 μM of the inhibitor (Fig. 3b).

**Fig. 4:**
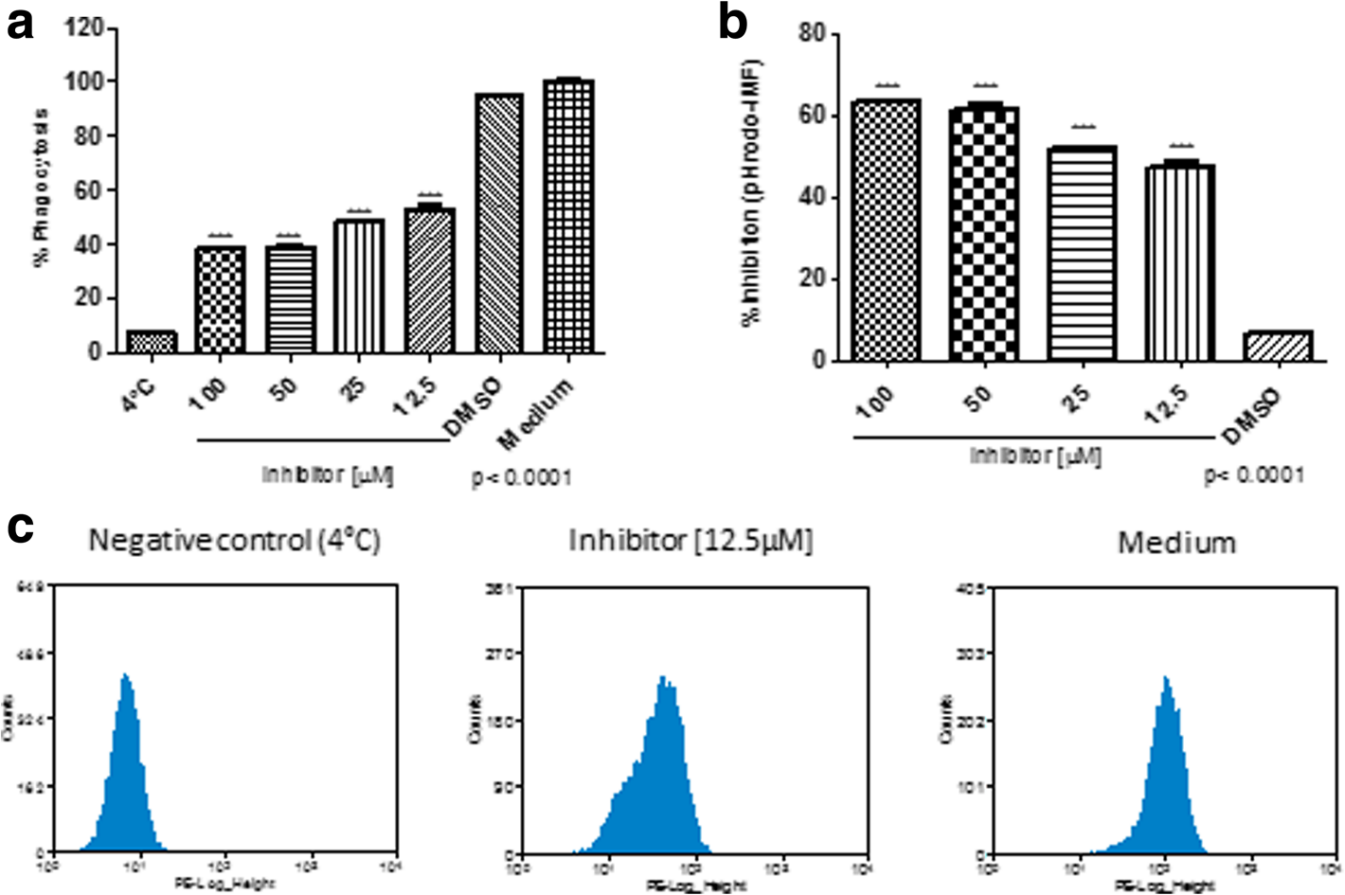
Inhibition of *E. coli* -containing phagosome formation by the 14-3-3 inhibitor in C6/36 HT cells. C6/36 HT cells were treated with 14-3-3 inhibitor in different concentrations and evaluated for the ability to form phagosome with pHrodo green *E. coli* bacteria. Phagosome formation (**a**, **b**) was significantly reduced by the treatment with inhibitors in dose-dependent manner (**c**) Representative histograms of phagosome formation by untreated C3/36 HT cells on ice (left, negative control, 4 °C), treated with 12.5 μM of 14-3-3 inhibitor (middle), and untreated C6/36 HT cells at 34 °C, in medium (right, positive control). Experiments were performed in triplicate and represented as the mean ± standard error, SE. Representative data from three independent experiments are shown

**Fig. 5:**
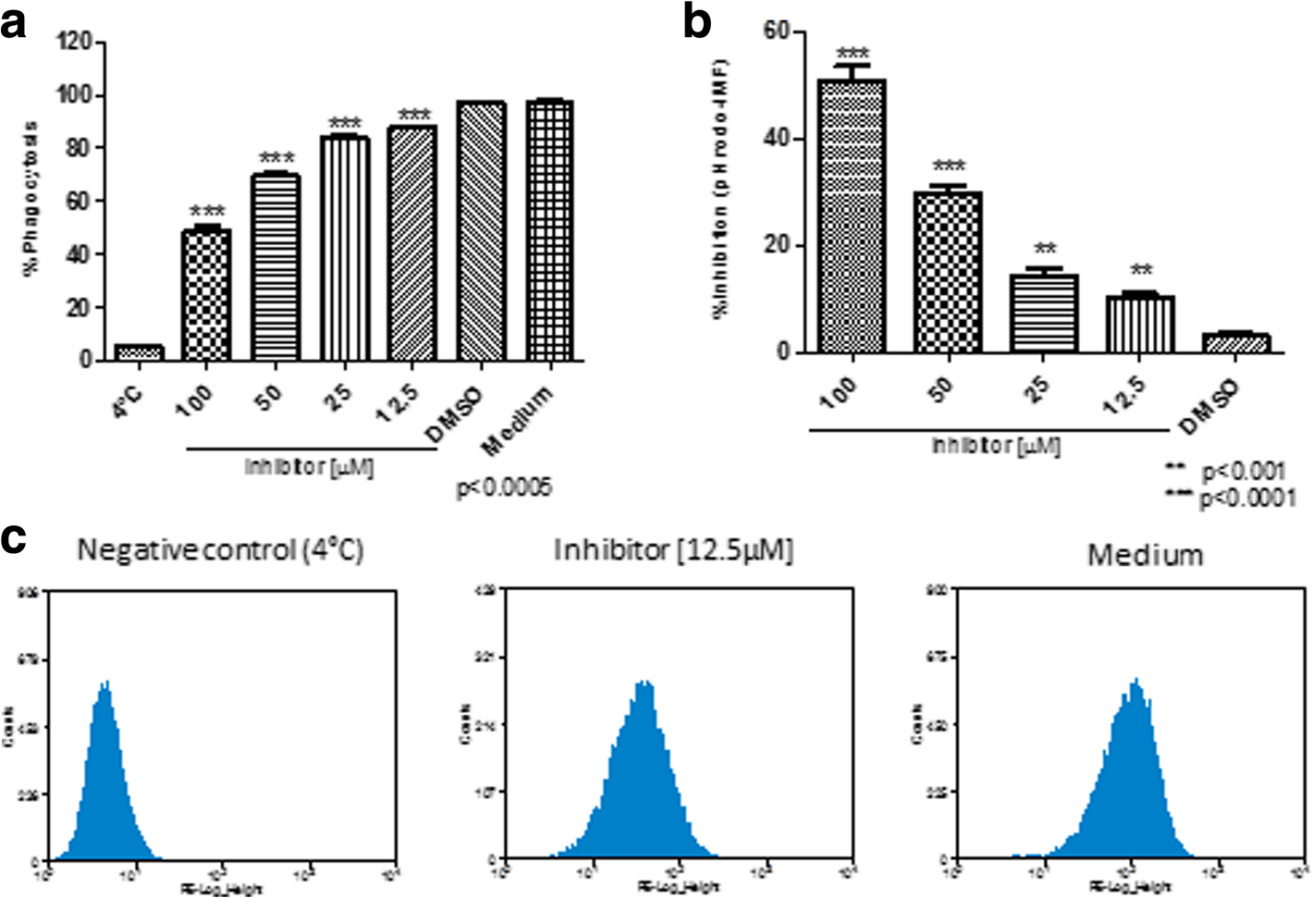
Inhibition of *S. aureus* -containing phagosome formation by the 14-3-3 inhibitor in C6/36 HT cells. C6/36 HT cells were treated with 14-3-3 inhibitor at different concentrations and evaluated for the ability to form phagosome with pHrodo green *S. aureus* bacteria. Again, phagosome formation (**a**, **b**) was significantly reduced by the 14-3-3 inhibitor in a dose-dependent manner. (**c)** Representative histograms of phagosome formation by phagosome formation by untreated C3/36 HT cells on ice (left, negative control), treated with 12.5 μM of 14-3-3 inhibitor (middle), and untreated C3/36 HT cells at 34 °C, in medium (right, positive control). Experiments were performed in triplicate and represented as the mean ± standard error, SE. Representative data from three independent experiments are shown

To further examine the participation of 14-3-3 in phagocytosis and phagosome maturation, we used pHrodo *E. coli* and *S. aureus* bioparticles in the presence of 14-3-3 inhibitor [31]. The pHrodo *E. coli* phagocytosis-phagosome formation decreased 50% (Kruskal-Wallis test: *χ* ^2^ = 13.04, *df* = 5, *P* = 0.011; t-test: *t* = 94.27, *P* < 0.0001) with 14-3-3 inhibitor concentrations of 12.5 and 25 μM, and 60% (Kruskal-Wallis test: *χ* ^2^ = 13.04, *df* = 5, *P* = 0.011; t-test: *t* = 37.40, *P* < 0.0001) with concentrations of 50 and 100 μM (Fig. 4). The pHrodo-*S. aureus* phagocytosis-phagosome formation was also reduced in the presence of 14-3-3 inhibitor; 10% (Kruskal-Wallis test: *χ* ^2^ = 13.52, *df* = 5, *P* = 0.0090; t-test: *t* = 8.315, *P* = 0.0011) at a concentration of 12.5 μM of inhibitor, 15% (Kruskal-Wallis test: *χ* ^2^ = 13.52, *df* = 5, *P* = 0.0090; t-test: *t* = 8.500, *P* = 0.001) at a concentration of 25 μM, 30% (Kruskal-Wallis test: *χ* ^2^ = 13.52, *df* = 5, *P* = 0.0090; t-test: *t* = 17.06, *P <* 0.0001) at a concentration of 50 μM and 50% (Kruskal-Wallis test: *χ* ^2^ = 13.52, *df* = 5, *P* = 0.0090; t-test: *t* = 16.40, *P* < 0.0001) at a concentration of 100 μM. These inhibitions were statistically significant at all concentrations (Fig. 5).

### 14-3-3 inhibitor effect on the cytoskeleton organisation

**Fig. 6:**
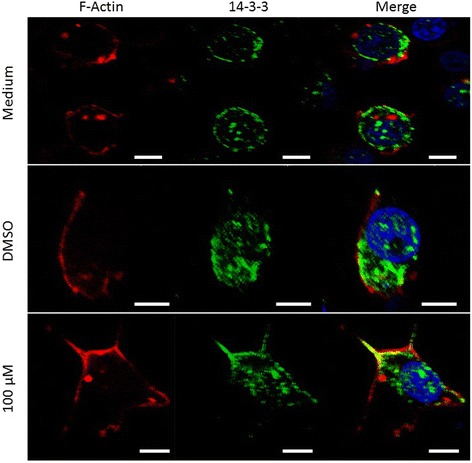
Effect of the 14-3-3 inhibitor on the cytoskeleton organisation of C6/36 HT cells. The intracellular distribution of 14-3-3 proteins (*green*), actin (*red*), and nuclei (*blue*) on C6/36 HT cells treated with 14-3-3 inhibitor, DMSO and medium were visualised by Immunofluorescence. In medium or DMSO treated cells, 14-3-3 proteins were present mainly in the cytoplasm and did not co-localized with F-actin in the cortex. In contrast, cells treated with 14-3-3 inhibitors underwent morphologic changes, e.g. elongation through reorganisation of F-actin and colocalizing with 14-3-3 proteins. *Scale-bars*: 5 μm

To investigate the role of 14-3-3 proteins in actin cytoskeleton organisation, we assessed the expression pattern of actin and 14-3-3 in C6/36 HT cells in the presence or absence of 14-3-3 inhibitor. In medium and DMSO control cells, F-actin was localised mainly at the cell cortex, and 14-3-3 was present in the cytoplasm and near the cell membrane. At 120 min after treatment with 14-3-3 inhibitor, cells became elongated (prismatic), actin reorganised and co-located with 14-3-3 in filopodia-like structures (Fig. 6).

### DsiRNAs mediated 14-3-3 silencing on C6/36 HT and Aag-2 cells

**Fig. 7:**
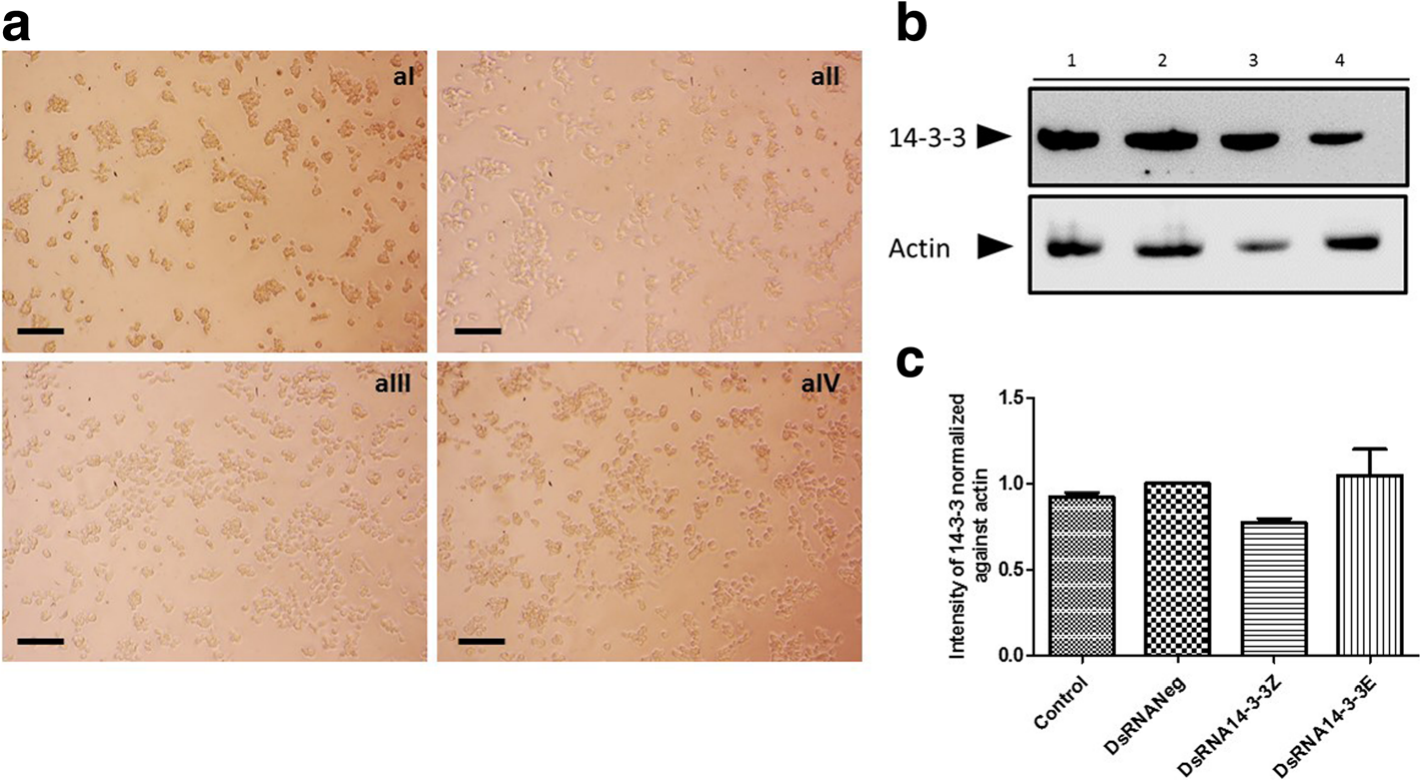
DsiRNAs effect on 14-3-3ε and 14-3-3ζ expression, in C6/36 HT cells. **a** C6/36 cells were double transfected with DsiRNA targeting 14-3-3, and expression of 14-3-3Ɛ and 14-3-3ζ proteins was assessed by Western blot. Optical microscopy images of C6/36 HT cells treated with (**aI**) DsiRNA 14-3-3Ɛ and (**aII**) DsiRNA14-3-3ζ, (**aIII**) DsiRNANeg and (**aIV**) DsiRNA control (Mock). (**b)** 14-3-3 protein expression on C6/36 HT cells treated with control (Mock) (Lane 1), DsiRNANeg (Lane 2), DsiRNA14-3-3Ɛ (Lane 3) and DsiRNA14-3-3ζ (Lane 4). **c** The expression of 14-3-3 proteins normalised against actin. *Scale-bars*: 100 μm

We attempted to investigate the role of 14-3-3 proteins in phagocytosis in *Aedes* spp. cell lines by “knockdown” of genes with DsiRNAs. Double transfection with DsiRNAs onto C6/36 HT cells did not elicit any major morphological changes compared with C6/36 HT cells transfected with controls (Fig. 7a) and no change in expression of 14-3-3ζ, and 14-3-3ε isoform proteins (*P* > 0.05) was observed (Fig. 7b, c).

**Fig. 8:**
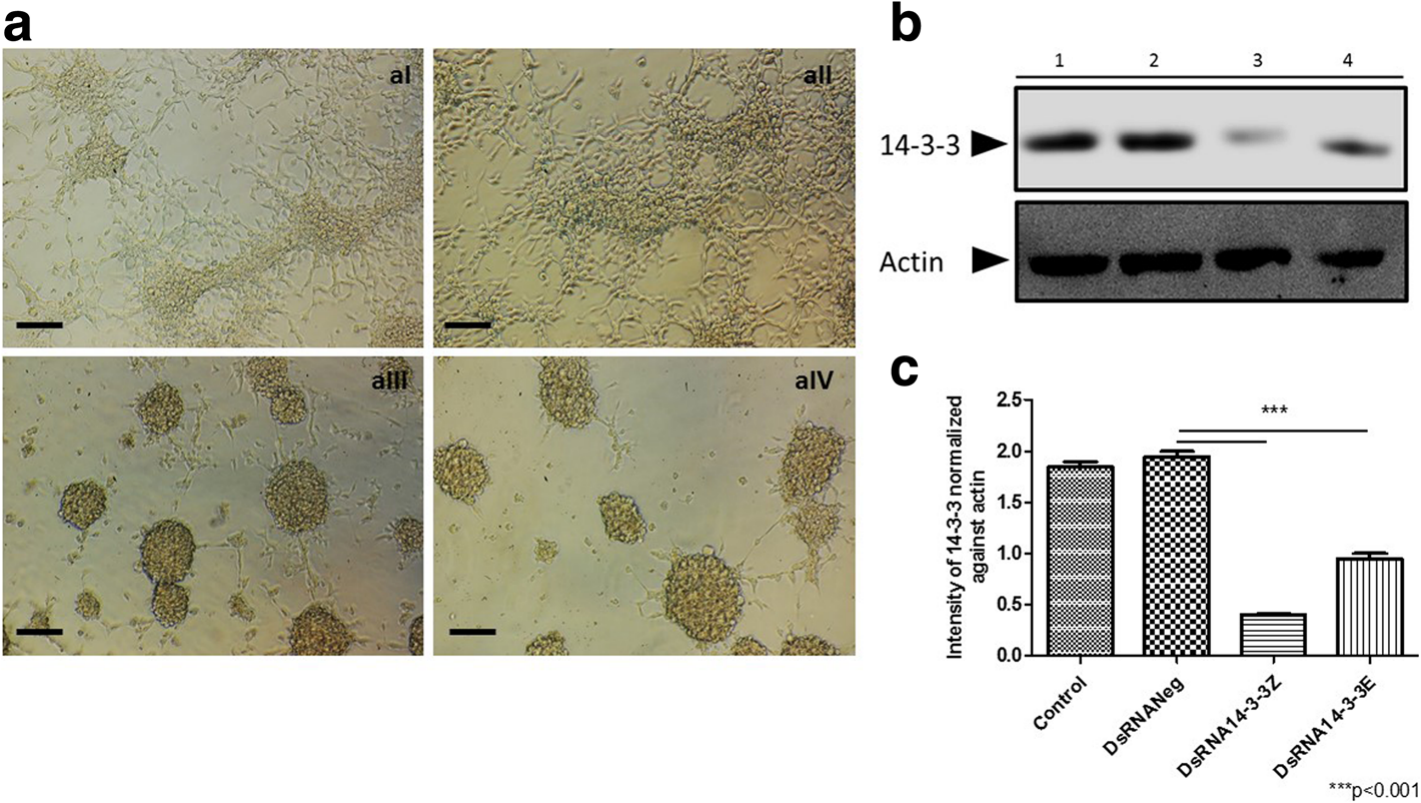
DsiRNAs mediated silencing of 14-3-3ε and 14-3-3ζ expression on Aag-2 cells. Aag-2 cells were double transfected with DsiRNA targeting 14-3-3, and expression of 14-3-3Ɛ and 14-3-3ζ proteins was assessed by Western blot. **a** Optical microscopy images of Aag-2 cells treated: (**aI**) DsiRNA control (Mock), (**aII**) DsiRNANeg, (**aIII**) DsiRNA14-3-3ζ and (**aIV**) DsiRNA 14-3-3Ɛ. **b** 14-3-3 protein expression on Aag-2 cells treated with control (Mock) (Lane 1), DsiRNANeg (Lane 2), DsiRNA14-3-3Ɛ (Lane 3) and DsiRNA14-3-3ζ (Lane 4). **c** The expression of 14-3-3 proteins normalised against actin. DsiRNAs successfully knockdown the expression of both 14-3-3ε and 14-3-3ζ in Aag-2 cells. *Scale-bars*: 100 μm

In contrast, Aag-2 cells transfected with DsiRNA14-3-3ε and DsiRNA14-3-3ζ dramatically underwent changes in their morphology from monolayer surface to a rounded cluster of cells, which has been described only in stress conditions (Fig. 8a) [32–34]. Moreover, the expression of both 14-3-3ε and 14-3-3ζ proteins was significantly reduced to 50 and 75%, respectively (t-test: *t* = 30.75, *P* = 0.0011) (Fig. 8b, c).

The effect to 14-3-3ε and 14-3-3ζ silencing showed the impaired phagocytosis-phagosome formation of pHrodo green *E. coli* in Aag-2 cells.

**Fig. 9:**
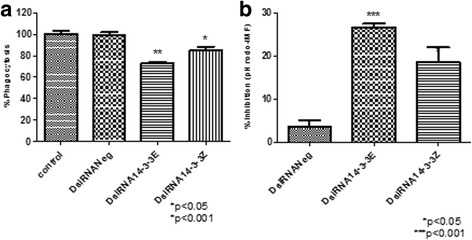
14-3-3ε and 14-3-3ζ are required for a normal phagocytosis in Aag-2 cells. **a** Aag-2 cells were double transfected with control (lipofectamine alone), DsiRNANeg, DsiRNA14-3-3Ɛ, and DsiRNA14-3-3ζ and phagosome formation of pHrodo *E. coli* was evaluated. The silencing of either 14-3-3Ɛ or 14-3-3ζ in Aag-2 cells resulted in significant reduction of phagosome formation (**a**), percentage inhibition of phagocytosis (pHrodo-IMF) on the C6/36 HT silencing comparated with the control (**b**). Representative data from three independent experiments are shown. Experiments were performed in triplicate and represented as the mean ± standard error, SE

Finally, we examined whether DsiRNA mediated silencing for 14-3-3ε and 14-3-3ζ affects the phagocytosis-phagosome formation of pHrodo *E. coli* in Aag-2 cells. We observed a significant reduction on phagocytosis-phagosome formation of pHrodo *E. coli* of 30% (t-test: *t* = 12.72, *P* = 0.0061) and 20% (t-test: *t* = 28.76, *P* = 0.0012) for 14-3-3ε and 14-3-3ζ, respectively (Fig. 9). Taken together, DsiRNA treatment effectively reduced the expression of 14-3-3 scaffold proteins, which led to significant reduction of the phagocytosis-phagosome formation of bacteria.

## Discussion

The members of the 14-3-3 family are 28–30 kDa acidic proteins highly conserved in a wide range of organism and tissues [35]. The 14-3-3 proteins interact as adapters, activators and repressors with several other protein partners, regulating signalling pathways in a vast array of processes such as apoptosis, cell signalling and cytoskeletal organisation [11, 36, 37].

Previously, two isoforms of 14-3-3ε and 14-3-3ζ have been shown to be present in insects such as *Drosophila*, *B. mori* and *Ae. aegypti* [9, 12, 13, 38]. In this work, we documented the expression and function of *Aeae*14-3-3ε and *Aeae*14-3-3ζ in C6/36 HT cells derived from *Ae. albopictus* and Aag-2 cells derived from *Ae. aegypti* as model systems from insect vectors of human diseases. We found that 14-3-3 proteins were localised in the cytoplasm in *Aedes* spp. cells in accordance to those observed in *Ae. aegypti* midguts [13]. This cellular distribution is similar to those observed in cells of several tissues of other insects, such as *B. mori* [38] and *Pieris rapae* [36]. This is important since on the isoforms that are present in different cellular locations under certain cellular conditions [29].

To investigate functions of 14-3-3 in C6/36 HT cells, we used a membrane-permeable specific inhibitor, which disrupts the formation of the functional complex through interactions with their protein partners [21, 22]. 14-3-3 inhibitor had not impacted on the viability of mosquito cells in the dose range tested (90% viability after 180 min), indicating that the observed functional effect in our study was not the result of cell damage. The 14-3-3 inhibitor-induced cytoskeletal reorganisation on C6/36 HT cells which led to changes in cell shape and co-localization of 14-3-3 proteins with F-actin in filopodium-like structures (Additional file 1: Fig. S1, Additional file 2: Fig. S2). In *Drosophila*, 14-3-3 proteins have been suggested to interact with several regulators of the actin cytoskeleton and, especially, the actin depolymerising factor cofilin, which is required for the efficient phagocytosis of bacteria [3]. Besides the interaction of 14-3-3 proteins with proteins located on actin filaments, such as cofilin, interactions with other proteins has also been documented with its regulatory kinase, LIM-domain protein kinase 1 [37] and other proteins involved in attachment of the cytoskeleton to the plasma membrane and to the extracellular matrix [39, 40]. In mice astrocytes, the presence of 14-3-3γ was observed to be close to actin filaments as ischemia and apoptosis induced changes in the binding of 14-3-3 to F-actin [40]. Proteomic experiments in mammalian cells provide further evidence of 14-3-3 interaction with actin [15, 41]. Together, these data suggest that 14-3-3 is involved in F-actin dynamics, probably interacting with actin-binding proteins.

Phagocytosis is a rapid-acting immune mechanism of insect haemocytes representing a primary defence line that limits microbial infection [42–44]. A role of 14-3-3 proteins in phagocytosis in *Drosophila* was already documented [3, 9, 45]. C6/36 HT is a plasmatocyte-like cell line derived from *Ae. albopictus* that has been used as an experimental model for phagocytosis [46, 47]. We observed the reducing on *S. typhimurium* and *E. coli* uptake by C6/36 HT cells under treatment with the 14-3-3 inhibitor, indicating that 14-3-3 proteins participate in the internalisation (uptake) in the phagocytosis of Gram-positive and Gram-negative bacteria. Considering that diminution of phagocytosis was indistinct for both Gram-positive and Gram-negative bacteria, the mechanism is probably independent of the receptor, and 14-3-3 proteins play a role in phagocytosis in a step after recognition of the target particle. Together, these results showed that 14-3-3 inhibitor is efficient in blocking the function of 14-3-3 proteins in mosquito C6/36 HT cells and causes the inhibition of phagocytosis of bacteria, indicating the participation of this protein in phagocytosis.

The exocyst complex is a molecular complex required for endo- and exo- cytosis, which is implicated in phagocytosis on *Drosophila*. The exocyst complex formation is an important step during phagosome biogenesis through the formation of the early phagosome [48]. We used pHrodo *E. coli* and *S. aureus* bacteria as useful markers of phagolysosome development because they become fluorescent when they are in the acidic environment of the phagolysosome [9, 28, 31, 49]. In this study, C6/36 HT cells treated with the 14-3-3 inhibitor showed a significant decrease in the percentage of phagocytosis and phagolysosome formation (pHrodo bright cells), indicating the involvement of 14-3-3 proteins in phagosome maturation likely in the fusion of phagosome with lysosomes at late stage of phagocytosis [50, 51], in addition to involvement of 14-3-3 proteins at early stage of phagocytosis - bacterial uptake [48, 50]. These results are consistent with observations in haemocytes from *Drosophila* 14-3-3ɛ null mutants, where bacterial internalisation, phagosome formation and maturation were also disrupted [9]. One evidence of the 14-3-3 participation in phagocytosis was the identification of 14-3-3 protein in latex bead-containing phagosomes in J774 mouse macrophage-like cell line [51]. There is also evidence for both 14-3-3ε and 14-3-3ζ participation during phagocytosis of *E. coli* and *S. aureus* [3, 9]. Here, we extended the role of 14-3-3 proteins beyond phagocytosis as our results suggest that the functional impairment of 14-3-3 proteins led to defects in phagolysosome formation.

We provided an additional line of support for the role of 14-3-3 proteins in phagocytosis through silencing expression of 14-3-3 genes at the mRNA level using DsiRNAs. RNAi mechanism depends on a molecular pathway that is triggered by long exogenous double-strand RNA (dsRNA) in the cell. Dicer-2 (Dcr2) recognises and cleaves the dsRNA into small interfering RNAs (siRNAs), which usually 21 bp initiating the RNAi pathway. The siRNA associated with Dcr2 are loaded into a multi-protein RNA-induced silencing (RISC), where it is processed (cleaved) [52–54]. We used a 27-mer instead of 21-mer of siRNAs and took advantage of the link between Dicer and RISC systems for silencing 14-3-3ε and 14-3-3ζ transcripts, as this approach can boost silencing efficiency by ca. tenfold compared with traditional siRNAs. We found very different outcomes in C6/36 HT and Aag-2 cells regarding morphology and protein expression after silencing 14-3-3 proteins. Aag-2 cells [55], showed a significant decrease in the 14-3-3ε and 14-3-3ζ protein expression and marked morphological changes with 14-3-3 DsiRNA silencing, in contrast to C6/36 HT cells that did not show changes in morphology nor significant decrease in 14-3-3 protein production. It has been reported that C6/36 HT cells exhibited inefficient Dcr2 cleavage of long dsRNAs or DsiRNAs while those Aag-2 cells were capable of siRNA processing. The defective expression or function of Dcr2 in C6/36 HT cells might explain the ineffective silencing likely from preventing activation of the RNAi machinery [20, 56, 57].

It is important to note that there are some differences between C6/36 HT cells derived from *Ae. albopictus* larvae whole cells [58, 59] and Aag-2 derived from *Ae. aegypti* embryonic cells [60]. In addition, the C6/36 HT cells have been adapted to grow at 34 °C (they grew at 28 °C when first isolated) [61, 62]. Nevertheless, filopodia-like structures have been observed in C6/36 HT cells that were infected with Japanese encephalitis virus (grew at 28 °C) [63]. In addition, the Aag-2 cells grew as attached fibroblast-like cells in monolayer [32] and tended to aggregate especially when cultured for a long term [32] or infected with Rift Valley fever virus [64]. The similar aggregate was likely observed in the Aag-2 cells transfected with DsiRNA in our study.

In summary, DsiRNA treatment effectively decreased 14-3-3ε and 14-3-3ζ scaffold protein expression in Aag-2 cells, which resulted in cytoskeleton reorganisation and decreased phagocytosis and phagosome maturation. Our results demonstrated the involvement of 14-3-3 proteins in phagocytosis, a central mechanism of innate immune responses in mosquitoes. As DsiRNA mediated silencing 14-3-3ε had a bigger impact compared to 14-3-3ζ, further investigations are needed to detail differential molecular and cellular mechanisms for 14-3-3ε and 14-3-3ζ, including the identification of proteins partners connecting with the actin cytoskeleton and signalling pathways involved.

## Conclusions

We conclude that 14-3-3ε and 14-3-3ζ proteins are essential for phagocytosis of Gram-positive and Gram-negative bacteria in the Aag-2 (*Ae. aegypti*) and C6/36 (*Ae. albopictus*). The inhibition of 14-3-3ε and 14-3-3ζ, affected cell morphology, phagocytosis, as well as the phagosome formation, presumably by modifying cytoskeletal remodelling process.

## Additional files

Additional file 1: Fig. S1. Phagocytosis of FITC-*E. coli* on C6/36 HT cells. The intracellular distribution of FITC-*E. coli* (green) and nuclei (blue) on C6/36 HT cells at the beginning of incubation with bacteria (T0) and after 30 min at 34 °C in medium alone (30 min) were visualised by immunofluorescence. (TIFF 259 kb)

Additional file 2: Fig. S2. Effect of the 14-3-3 inhibitor on the cytoskeleton organisation of C6/36 HT cells. The intracellular distribution of 14-3-3 proteins (red) and nuclei (blue) on C6/36 HT cells cultured in medium alone (MEDIUM), vehicle (DMSO) and 14-3-3 inhibitor (INHIBITOR) were visualised by immunofluorescence. (JPEG 78 kb)

## Abbreviations

DMSO: Dimethyl sulfoxide
DsiRNAs: Dicer-substrate siRNAs
dsRNA: Double-strand RNA
FITC: Fluorescein
GFP: Green fluorescent protein
HBSS: Hank’s balanced salt solution
INH: 14-3-3 inhibitor
MEM: Minimum essential medium
RNAi: Ribonucleic acid interference
RT-PCR: Reverse transcription polymerase chain reaction
siRNAs: Small interference RNA
