# Supplementary figures and images for: Participation of 14-3-3ε and 14-3-3ζ proteins in the phagocytosis, component of cellular immune response, in Aedes mosquito cell lines
Source: Parasit Vectors. 2017 Aug 1;10:362. doi: 10.1186/s13071-017-2267-5 (PMC5540338; doi:10.1186/s13071-017-2267-5)

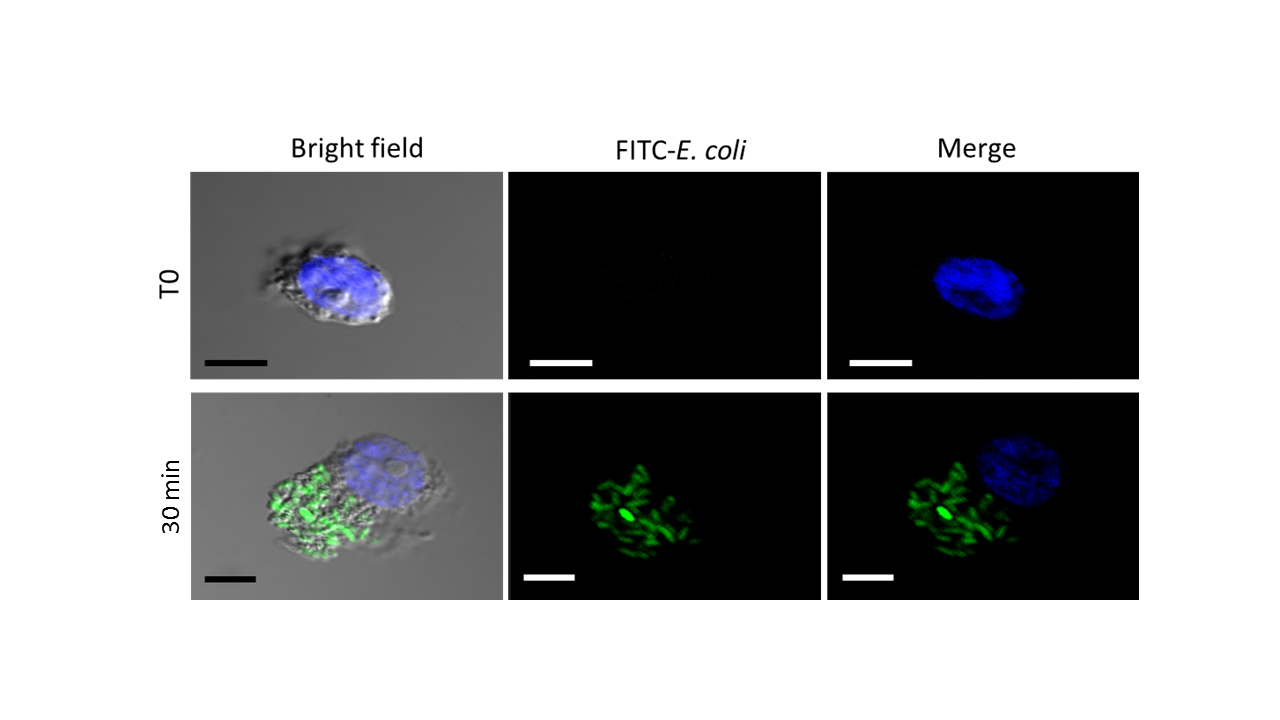

Supplement: Supplementary file 1 — Phagocytosis of FITC-E. coli on C6/36 HT cells. The intracellular distribution of FITC-E. coli (green) and nuclei (blue) on C6/36 HT cells at the beginning of incubation with bacteria (T0) and after 30 min at 34 °C in medium alone (30 min) were visualised by immunofluorescence. (TIFF 259 kb) [file 13071_2017_2267_MOESM1_ESM.tif]

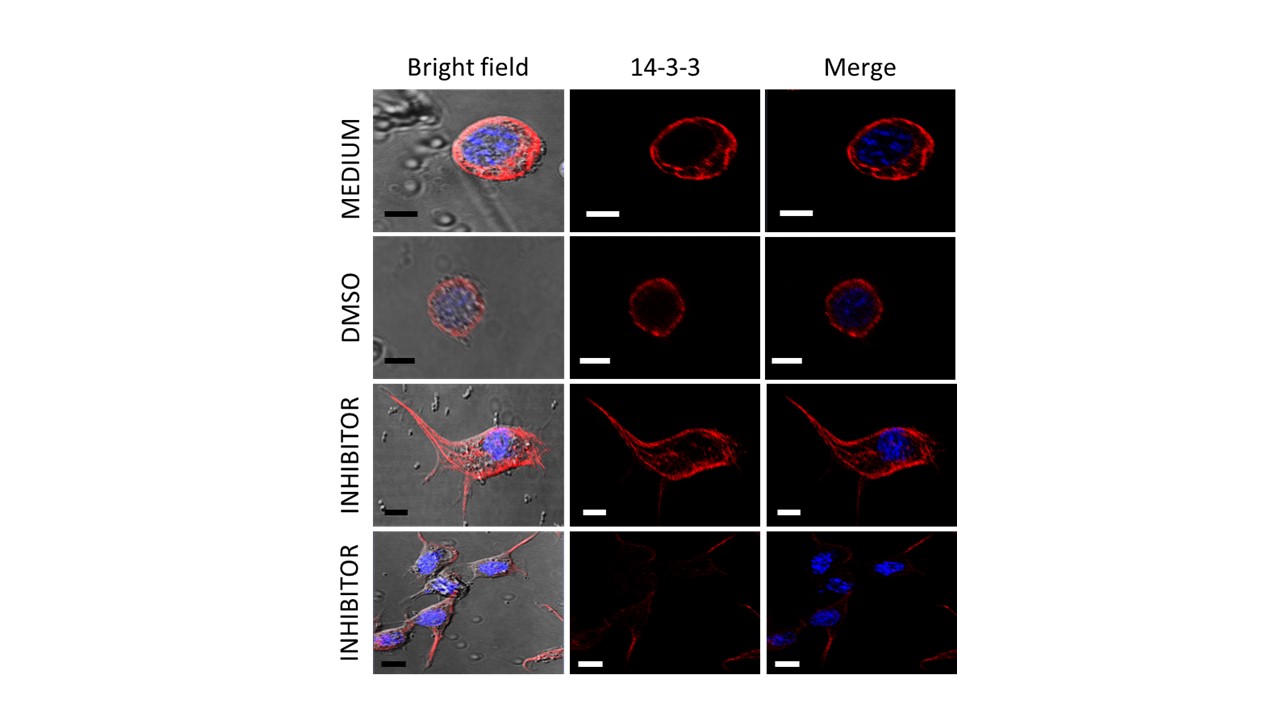

Supplement: Supplementary file 2 — Effect of the 14-3-3 inhibitor on the cytoskeleton organisation of C6/36 HT cells. The intracellular distribution of 14-3-3 proteins (red) and nuclei (blue) on C6/36 HT cells cultured in medium alone (MEDIUM), vehicle (DMSO) and 14-3-3 inhibitor (INHIBITOR) were visualised by immunofluorescence. (JPEG 78 kb) [file 13071_2017_2267_MOESM2_ESM.jpg]
